# Supplementary material for: Antibody levels and protection after Hepatitis B vaccine in adult vaccinated healthcare workers in northern Uganda
Source: PLoS One. 2022 Jan 21;17(1):e0262126. doi: 10.1371/journal.pone.0262126 (PMC8782524; doi:10.1371/journal.pone.0262126)
Supplement: S1 Appendix — (PDF) [file pone.0262126.s001.pdf]

Prevalence of Hepatitis B virus protective immune response among vaccinated healthcare personnel in selected regional referral hospitals in Uganda

Questionnaire code: ____ ____

Sociodemographic characteristics

1. Sex   Male ☐   Female ☐

2. Age (at last birthday) ______________________________

3. Specialty/trainee position *(Tick ONLY one)*

**Specialty / Trainee position**	**Yes/No** *(Tick)*	**Years spent working at this position** *(months or years)*
Medical officer		
Clinical officer		
Pharmacist		
Pharmacy tech		
Nursing officer		
Laboratory technician		
Midwife		
Nursing assistant		
Intern doctor		
Intern pharmacist		
Intern nurse		
Intern pharmacist		
Others specify		

Hepatitis B virus risk and vaccination

4. Have you had any of the following during the last three months *(Tick ALL that apply)*

**Exposure**	**Yes**	**No**
Needle prick injury		
Blood splash		
Blood transfusion		
Injection (I.M/I.V)		
Conducted surgical operation		
Had a tattoo on your skin		
Undergone an a surgical operation		
Others specify,		

MAKERERE UNIVERSITY SCHOOL OF BIOMEDICAL SCIENCES APPROVED VALID UNTIL 29 JUL 2021 RESEARCH & ETHICS COMMETTEE P. O. BOX 7072, KAMPALA

5. Have you ever tested for hepatitis B virus infection? Yes ☐ No ☐

6. Have you had hepatitis B virus vaccine before? Yes ☐ No ☐

If yes to question 6, answer questions 7-10

7. When did you receive the last dose of hepatitis B vaccine (day/month/year)_____________________

8. What was your age at the time you received the hepatitis B vaccine___________

9. How many doses of the vaccine did you get _______________

10. Did you complete all the hepatitis vaccine series (3 doses)? Yes ☐ No ☐

11. In what time interval (in months) did you receive the vaccine? *(Tick only ONE)*

**Time interval** *(Months)*	**Yes**	**No**
0, 1, 6		
0, 3, 6		
0, 1, 2		
0, 2, 4		
Do not recall		

12. If you did not complete all the hepatitis vaccine series, what was the reason? *(Tick ALL which apply)*

**Reason for not completing hepatitis B vaccine series**	**Yes**	**No**
The vaccine ran out of stock		
Lacked money to pay for the vaccine		
I lost interest in completing the dose		
Others specify,		

MAKERERE UNIVERSITY
SCHOOL OF BIOMEDICAL SCIENCES
APPROVED
VALID UNTIL
29 JUL 2021
RESEARCH & ETHICS COMMETTEE
P. O. BOX 7072, KAMPALA

13. Did you perform hepatitis antibody titre test after completing the hepatitis B vaccine series

Yes ☐ No ☐

14. What was the concentration of hepatitis B antibodies found ____________mIU/mL

15. Have you received a booster dose of hepatitis B vaccine? Yes☐ No☐

16. If yes to 12, when did you receive the booster dose of hepatitis B vaccine? __________

17. In your facility, what factors would affect the hepatitis B vaccine efficacy (how well the vaccine will work if given)? *(Tick ALL that apply)*

**Factor(s)**	**Yes**	**No**
Power fluctuations affecting hepatitis vaccines in storage		
Mechanical problems affecting the fridge		
People do not complete full vaccine series (3doses)		
Type of vaccine given (plasma derived or recombinant)		
Vaccine over staying in storage		
Do not know		
Others specify,		

THE SECTION TO BE FILLED BY RESEARCHER

18. Hepatitis screening results *(Tick ONE which apply)*

**Screening test**	**Results**	
	**Positive**	**Negative**
HEPATITIS B SURFACE ANTIGEN (HBsAg)		
HEPATITIS B SURFACE ANTIGEN ANTIBODY (HBsAb)		
HEPATITIS B ENVELOPE ANTIGEN (HBeAg)		
HEPATITIS B ENVELOPE ANTIBODY (HBeAb)		
HEPATITIS B CORE ANTIBODY (HBcAb)		

19. ANTIBODY TITRE CONCENTRATION................................mIU/mL

MAKERERE UNIVERSITY
SCHOOL OF BIOMEDICAL SCIENCES
APPROVED
VALID UNTIL
29 JUL 2021
RESEARCH & ETHICS COMMETTEE
P. O. BOX 7072, KAMPALA
