# Supplementary material for: Antibody levels and protection after Hepatitis B vaccine in adult vaccinated healthcare workers in northern Uganda
Source: PLoS One. 2022 Jan 21;17(1):e0262126. doi: 10.1371/journal.pone.0262126 (PMC8782524; doi:10.1371/journal.pone.0262126)
Supplement: Attachment — Submitted filename: Comment to the authors.docx [file pone.0262126.s002.docx]

Firstly, I would like to congratulate the authors for addressing such an important public health topic with a relevant methodology. Generally, the paper *is* well written and structured. However, further efforts are required to make the study more attractive to the readers. I have provided and raised numerous comments and questions on the manuscript that has to be addressed by the authors. These are:

1. In my opinion, it is better if you modify your title to make it easily understandable, attention grasping and in line with your study objectives.
2. Your sample size should be mentioned in the abstract section in between line number 28 and 29.
3. You put too many information under abstract section. Results that answer your objectives are enough.
4. Introduction section

You stated that “However, the vaccine may not provide protection from exposure to hepatitis B virus later on in life due to waning of immune memory over time (3).” This idea needs a strong scientific evidence and it should be cited with additional and credible source to convince the readers.

5. Method and materials

- The tittle in line number 105 should be “Method and Materials”, instead of method.
- Your sampling technique is not stated.
- No exclusion or inclusion criteria?
- The following terms should be defined under method section. Otherwise, it will be difficult to understand the results.
- Hepatitis B surface antigen (HBsAg), Hepatitis B surface antibody (HBsAb), Hepatitis B 130 envelop antigen (HBeAg), Hepatitis B envelop antibody (HBeAb), and Hepatitis B core antibody 131 (HBcAb).”
- Line number 148 “Hepatitis B antibody levels ≥ 10mIU/mL were considered protective.” The recommendation for this cut off value should be cited.

Results

- Which one is your research objective? The first or the second sentence? Is immune development status due to vaccination and natural infection the same?

“*Where 93.6% (249/266, 95%CI: 89.9-96) had protective immune response (> 10mIU/mL) against hepatitis B virus*.”

*“Overall, we found that the majority, 72% (216/300) of the study participants had protective hepatitis B immunity 192 (≥10mIU/mL) due to hepatitis B vaccine.”*

- In line number 192and 193 you said that “due to hepatitis B vaccine” and “due to a natural infection”. These should be defined under definition of terms of the methodology part and readers can understood easily it meaning.
- You have duplication of ideas in between line number 184 up to 187.
- All the points in table 2 are described under the paragraphs above. So, what is the importance of this table? I think it is duplications of efforts and the interpretations in table 2 are ideas to be mentioned under definition of terms/operational definition. You can simply remove the last column (frequency) and put it under method section as operational definition or you can modify it.
- In line number 206 and 207, you stated “Factors associated with hepatitis B virus immune response among 207 healthcare workers in Gulu and Lira Regional Referral hospitals”. It is too long and seems table tittle.
- There is no need to prepare different tables for bivariate and multivariate regressions models. Please merge table 3 and 4.
- In multivariate analysis, your two-by-two table did not fulfill the Chi-square assumption for regression. There are empty cell and your CI also includes 1, but your p value is less than 5%. How can it be possible? I don’t think so that you have a significant predictor.
- Your statement under table 4 is difficult to understand, for instance “were 7% times” and the likes. The paragraph from line number 119 up to 229 are also not easily understandable.
- Be consistent throughout. Example, for your dependent variable, use either “protective anti-HBs levels” or “hepatitis B immune response” throughout.
- In your recommendation, you did not talk about the factors of protective anti-HBs levels. Is that not your study objective from the outset or not?
- Grammar and punctuation errors are her and there in the paper and sometimes your idea is difficult to understand and not coherent. Please get assistance from someone who is fluent in English language and/or manuscript editing software’s.
