# Supplementary material for: Antibody levels and protection after Hepatitis B vaccine in adult vaccinated healthcare workers in northern Uganda
Source: PLoS One. 2022 Jan 21;17(1):e0262126. doi: 10.1371/journal.pone.0262126 (PMC8782524; doi:10.1371/journal.pone.0262126)
Supplement: Attachment — Submitted filename: Comments to authors_HBV in northern Uganda.docx [file pone.0262126.s003.docx]

General comments: I would like to thank the authors for this wonderful article. It addresses a public health concern that is to some extent ignored in many low resource settings. The article also builds on previous work undertaken in Uganda. The paper is well-written. Nonetheless, there are a few issues that the authors need to address to make the paper more scientifically informative.

1) Methods: Provide sufficient detail on the study setting. Where are the study facilities located? How are the staffing levels, cadres and services provided? Do the healthcare facilities provide Hepatitis B screening and management services?

2) Methods: Why was Mulago national referral hospital chosen for the pre-test? Is Mulago hospital and the two study healthcare facilities comparable?

3) A section on quality assurance and control for both serological testing and interviews with health workers is missing. Were the research assistants trained?

4) Discussion: Generally, the authors need to strengthen the discussion. Whereas they have attempted to provide implications for the findings, there is no explanation for most of the findings. For example, what explains the high proportion of healthcare workers with detectable anti-HBs?

5) Discussion: The authors point out that there was an improvement in the hepatitis B vaccination rate compared to a previous study by Ssekamatte et al. Both studies are recent and were conducted in Uganda. What explains the improvement? Are there any contextual differences between healthcare facilities in Wakiso and Lira and Gulu hospitals? What explains the high vaccine completion rates in Gulu and Lira referral hospitals? Remember that regional referral hospitals also have treatment centres for Hepatitis B.

6) Discussion: The authors mentioned that ‘’ While only three of the healthcare workers had received a booster dose of the hepatitis B vaccine, we found no significant difference in the presence of protective immune response between individuals who received a booster dose and those who did not’’. However, the authors do not provide an explanation to the findings. Why is it so?

7) Details of the ethics approval are lacking. Kindly provide information on the ethics clearance for the current study.
